# Validation of an HPLC Method for Pretreatment of Steviol Glycosides in Fermented Milk

**DOI:** 10.3390/foods10102445

**Published:** 2021-10-14

**Authors:** Jin-Man Kim, Jong-Ho Koh, Jung-Min Park

**Affiliations:** 1 Department of Food Marketing and Safety, Konkuk University, Seoul 05029, Korea; jinmkim@konkuk.ac.kr; 2 Department of Bio-Food Analysis, Bio-Campus, Korea Polytechnic College, Nosan 32940, Korea; kohjh100@naver.com

**Keywords:** pretreatment, HPLC, steviol glycosides, dairy products, fermented milk

## Abstract

Steviol glycosides are used in food and beverages worldwide as natural sweeteners, serving as a low-calorie sugar substitute. The acceptable daily intake of steviol is 0–4 mg/kg body weight. The rising demand for dairy products has led to a corresponding increase in the use of steviol glycosides in such products. Therefore, it is important to analyze the levels of steviol glycosides in dairy products. Dairy products have high fat contents and unique emulsion characteristics, conferred by a mixture of fat globules, casein micelles, whey proteins, and numerous other small molecules. These characteristics may interfere with the estimation of steviol glycoside levels; therefore, dairy samples require pretreatment. We aimed to develop an objective test for measuring the levels of steviol glycosides through the development of an efficient pretreatment method. In this study, the steviol glycoside content in dairy products was evaluated by using various methods, and an optimal pretreatment method was determined. We used high-performance liquid chromatography to assess the selectivity, linearity, limit of detection, limit of quantification, accuracy, precision, and recovery rate. Calibration curves were linear in the range of 1–50 mg/kg, with a coefficient of determination of ≥0.999. The limit of detection and limit of quantification were in the ranges of 0.11–0.56 and 0.33–1.69 mg/kg, respectively. The relative standard deviation (%) represents the precision of a measurement. The RSD relative standard deviationof recovery varied between 0.16% and 2.83%, and recovery of the analysis varied between 83.57% and 104.84%. These results demonstrate the reliability of the method for measuring the steviol glycoside content. This method can be used for the simple pretreatment of steviol glycosides and can provide an accurate determination of steviol glycoside content in emulsified food matrices, such as dairy products.

## 1. Introduction

*Stevia rebaudiana* Bertoni is a potential source of natural non-caloric sweeteners that may serve as substitutes for synthetic sweeteners. Its use is approved in Brazil, Argentina, Paraguay, China, Korea, and Japan [1]. The high sweetness of its components makes it an attractive substitute for sugar in the food industry [2]. *Stevia* also has hypotensive, hypoglycemic, anticarcinogenic, antioxidant, antimicrobial, anti-inflammatory, and antitumor activities [3,4]. The sweetening effect of *Stevia* is attributable to the fact that it contains glycosides of the aglycone steviol [5,6,7,8]. Approximately 40 steviol glycosides have been identified in the leaves of *S. rebaudiana* [9,10]. Moreover, this plant has a very high sweetness potential because of its high content of diterpene glycosides, such as stevioside; rebaudioside A, C, and D; and dulcoside A [1].

*S. rebaudiana* Bertoni is a sweet herb native to South America (Paraguay and Brazil). The main steviol glycosides are stevioside, rebaudioside A, rebaudioside C, and dulcoside A; and rebaudioside A and stevioside are the most abundant [11,12,13]. Crude extracts of *Stevia* have a bitter or licorice-like aftertaste, which can affect consumer liking. While stevioside is 250–300 times sweeter than sucrose, it has a bitter aftertaste; in contrast, rebaudioside A is 350–450 times sweeter than sucrose and does not have an aftertaste [14]. Therefore, rebaudioside A is the principal component in commercial *Stevia* extracts. Although safety evaluations have been conducted by multiple regulatory bodies, there is currently no consensus on the toxicological properties of *Stevia* extracts. Various steviol glycosides remain popular as low and no-calorie sweeteners; however, there are ongoing studies on their potential adverse health effects [15].

The Joint Expert Committee on Food Additives (JECFA) and the European Food Safety Authority have assigned an acceptable daily intake (ADI) of 0–4 mg steviol equivalents per kg body weight, which was implemented in December 2011 [16,17]. The use of steviol in various food categories, including flavored fermented milk products, ice cream, chocolate products, fine bakery wares, and other dairy products, is regulated. To comply with the directives of the regulatory agencies, a simple and specific analytical method is necessary for the qualitative and quantitative analysis of steviol glycosides in sweetened commercial stevia extracts and foods [18,19,20].

The concentration of steviol glycosides is determined by using multiple techniques, including high-performance thin-layer chromatography (HPTLC) [21], enzymatic hydrolysis [22,23], capillary electrophoresis [24], near-infrared spectroscopy, ion-exchange resin chromatography [25,26], LC/mass spectrometry [27], and high-performance liquid chromatography (HPLC) with UV detection [28,29,30,31]. These methods vary in their sensitivity, precision, and accuracy. However, as the FAO/WHO JECFA has recommended HPLC for determining the steviol glycoside content [32], this is considered the most appropriate method [33,34].

Milk is a unique colloidal dispersion with high stability. It is a highly heterogeneous matrix comprising fatty acids, including palmitic, oleic, and linoleic acid; fat-soluble vitamins; and a high level of proteins and carbohydrates. These constituents could interfere with the detection of steviol glycosides [35,36]; therefore, it is important to optimize the method of sample preparation for milk and milk-based products. As the concentrations of steviol glycoside additives in milk are usually very low [37], it is important to have an effective sample pretreatment method to remove its emulsion characteristics in order to extract steviol glycosides [38].

Many extraction solvents (acetonitrile, chloroform, and methanol) and methods (enzymatic extractions, supercritical fluid methods, microwaves, ultrasound followed by column purification, solvent–liquid–liquid extraction, ion exchange, membranes, nanofiltration, crystallization, and fractional distillation) are used for obtai ning steviol glycosides from *Stevia* leaves [39,40,41]. However, evaluating the steviol glycoside content remains difficult, especially in food and dairy products with high fat or protein content. There have been several studies analyzing steviol glycosides extracted from leaves; however, studies analyzing the steviol content in food are limited [42,43].

When milk is used as a matrix, particular attention should be paid to the sample preparation stage. As such, the proposed selective pretreatment method may be an attractive method, allowing optimal isolation and purification.

This study aimed to identify the optimal conditions for extracting steviol glycosides from fermented milk. Furthermore, we sought to validate the process by using an acidifying solvent pretreatment and analyze the extracts by using HPLC–UV/VIS. We aimed to define a methodology for measuring steviol glycosides in fermented milk, which will aid in developing methods for analyzing steviol content in other dairy products.

## 2. Materials and Methods

### 2.1. Reagents and Samples

Rebaudioside A (Reb A > 97%), rebaudioside B (Reb B > 92%), rebaudioside C (Reb C > 92%), rebaudioside D (Reb D > 94%), rebaudioside F (Reb F > 94%), dulcoside A (Dul A > 93%), rubusoside (RS > 91%), steviolbioside (SB > 90%), and stevioside (SV < 96%) were purchased from the LGC Group (Middlesex, UK) at a purity of 90–97%. Acetonitrile (ACN), methanol, and water (HPLC grade) were purchased from J.T. Baker (Phillipsburg, NJ, USA). Standard solutions of the nine steviol glycosides were prepared in 30% ACN, at a concentration of 500 mg/L. One bottle of non-fermented milk and eight bottles of fermented milk were purchased from a local market and stored at 4 °C.

### 2.2. Identification of the Optimal Pretreatment Method

**Figure 1:**
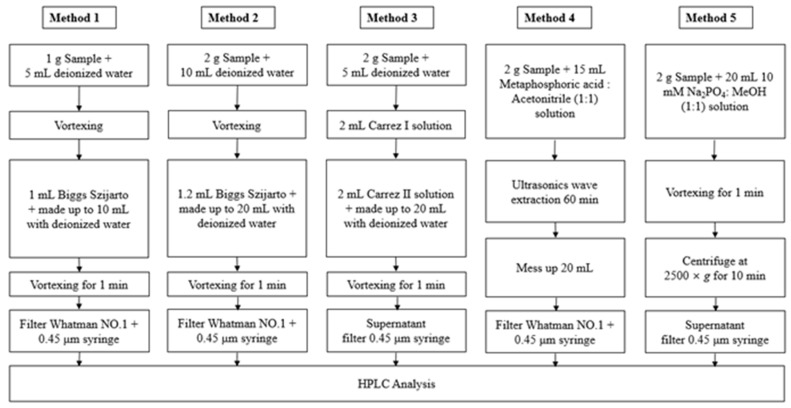
Comparison of the analytical steps required for determining steviol glycoside content in fermented milk products.

Among the methods established by the International Dairy Federation (IDF), two methods based on the Biggs–Szijarto method (using various proportions of phosphotungstic acid monohydrate, zinc acetate dihydrate, and acetic acid) were tested [44], along with two other methods. The first method used 1 mL Biggs–Szijarto solution and 10 mL deionized water (DW), while the second method used 1.2 mL Biggs–Szijarto solution and 20 mL DW. The third method used 2 mL Carrez reagent I and II and 20 mL DW with a modified food codex [45,46]. The fourth method used metaphosphoric acid and acetonitrile solution with a modified food codex for the acid hydrolysis of steviol glycosides [47]. The supernatant of each sample was filtered by using a 0.45 μm filter and used as the test sample. The fifth method used 2 g of the sample that was dissolved in 20 mL of 10 mM NaH_2_PO_4_:MeOH (1:1). The pH of the NaH_2_PO_4_ solution was adjusted to 2.6. Casein, which accounts for ~80% of the protein content of milk, is the major emulsification component of milk; therefore, it was removed to assist fat removal. Lowering the pH below 4.0 (the isoelectric point of casein) resulted in isoelectric precipitation. Methanol was added to increase the solubility of steviol. The sample was mixed well by vortexing for 1 min and centrifuged at 2,500× *g* at 10 °C for 10 min. Twenty milliliters of the solution was filtered, using a 0.45 μm filter, and used as the test sample. The optimal pretreatment method was selected from the five tested methods (Figure 1).

### 2.3. HPLC Analysis

HPLC was performed according to JECFA [32], using a Shimadzu HPLC system (Shimadzu Corporation, Kyoto, Japan) and ODS C18 columns (length: 250 mm; inner diameter: 4.6 mm; particle size: 5 μm) (Capcell Pak C18 MGII; Shiseido Co., Ltd., Tokyo, Japan) with a UV–VIS detector (210 nm). The mobile phase was a 32:68 (*v*/*v*) mixture of ACN and 10 mmol/L sodium phosphate buffer (pH 2.6) at a flow rate of 1.0 mL/min, and the column temperature was maintained at 40 °C. Standard steviol glycoside solutions were prepared at concentrations of 100–1000 mg/kg. The standard and sample solutions (20 µL) were injected into the HPLC system. Steviol glycosides were identified based on the retention times, using the standard mixture as a reference, and the peak areas were measured. Each solution was analyzed in triplicate, and the mean value was used for analysis.

### 2.4. Evaluation of Steviol Glycoside Content

To determine the steviol glycoside content, the reliability, consistency, and validation parameters were calculated. In agreement with the AOAC guidelines [48], the linearity, limit of detection (LOD), limit of quantification (LOQ), accuracy, and precision of each steviol glycoside were calculated. For this, 500 mg/L of standard was weighed and dissolved in 30% ACN to prepare a stock solution. The stock solution was diluted with HPLC water in the concentration range of 1–50 mg/kg for each glycoside. Each concentration was analyzed six times, using HPLC. The average values were used for plotting the standard curves.

Linearity was determined from the average coefficient of determination (r^2^), and calculations were performed by using a six-point standard curve in the concentration range of 1–50 mg/kg. The LOQ and LOD were estimated from the standard calibration curve, with signal-to-noise ratios (S/N) of 10 and 3, respectively.

The accuracy of the method was determined by using recovery tests with a commercial steviol glycoside mixture (dairy product). The commercial steviol glycoside mixture was spiked with 6.25, 12.5, and 25 mg/kg steviol glycoside. Each sample was analyzed in triplicate.

The recovery rate (%) of the analyte was calculated by using the following formula:

(1) $$A=\frac{\left(A1-A2\right)}{A3}\times 100$$

where *A*1 is the value obtained by using the steviol glycoside spiked standard solution, *A*2 is the value obtained when the standard solution is not used, and *A*3 is the amount of steviol glycoside spiked in the standard solution.

### 2.5. Monitoring Test

One sample of non-fermented milk and eight samples of fermented milk were tested for determining the steviol glycoside content.

### 2.6. Statistical Analysis

Each analysis was conducted at least thrice under each set of experimental conditions. Analysis of variance was performed by using Statistical Packaging for the Social Sciences (SPSS) for Windows (version 10.0). Duncan’s multiple-range test was used to compare the means of each treatment. A value of *p* < 0.05 was considered statistically significant. All data are expressed as the mean ± standard deviation (SD).

## 3. Results and Discussion

### 3.1. Optimization of the Pretreatment Conditions

**Figure 2:**
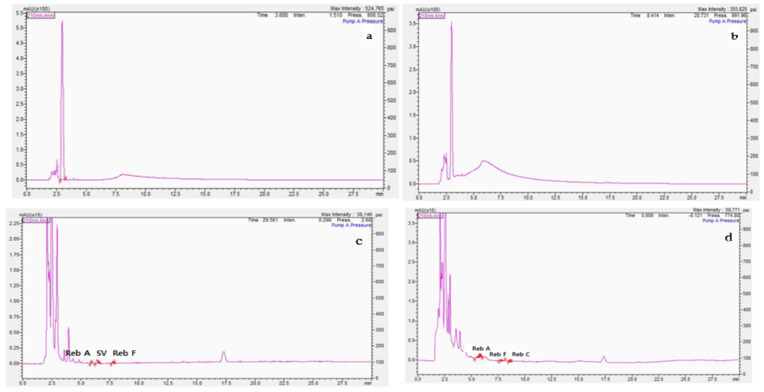

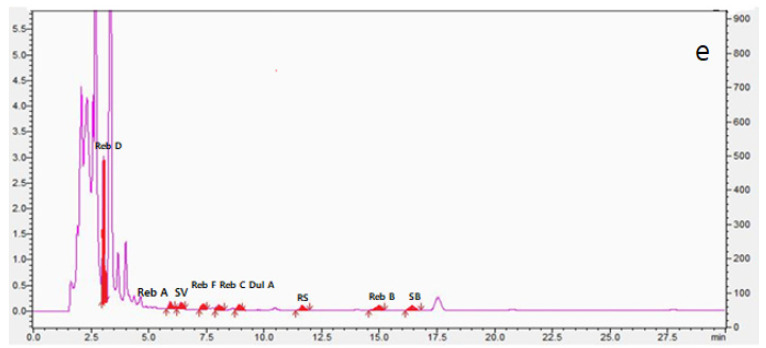
Chromatogram of the fermented-milk product spiked with steviol glycosides at a concentration of 500 mg/L (90–97% purity), following the various pretreatment methods. (**a**) First treatment, (**b**) second treatment, (**c**) third treatment, (**d**) fourth treatment, and (**e**) fifth treatment. Rebaudioside D (Reb D), rebaudioside A (Reb A), stevioside (SV), rebaudioside F (Reb F), rebaudioside C (Reb C), dulcoside A (Dul A), rubusoside (RS), rebaudioside B (Reb B), and steviobioside (SB).

In this study, five pretreatment methods for fermented milk samples were evaluated to select the best conditions for extracting steviol glycosides (Figure 2).

In the first method, all nine peaks could not be confirmed in the chromatogram. Preprocessing with the Biggs–Szijarto solution, which is an acidic solution, destroys the emulsification properties of milk. The sample peak obtained after this treatment overlapped with the solvent peak at a retention time of 2 min; therefore, the presence of steviol glycosides could not be ascertained, and no peaks corresponding to the samples could be confirmed (Figure 2a).

In the second method, when the samples were preprocessed, a transparent solution was obtained after layer separation and filtration. However, all nine peaks could not be identified in the chromatogram, thus indicating that the emulsification properties were not completely eliminated (Figure 2b).

In the third method, a transparent solution was obtained after layer separation and filtration. However, only three (Reb A, SV, and Reb F) of the nine steviol glycosides were identified in the chromatogram (Figure 2c).

In the fourth method, the fermented milk sample was processed by using metaphosphoric acid and acetonitrile (1:1, *v*/*v*) for emulsion disruption. Three steviol glycosides (Reb A, Reb F, and Reb C) were identified in the chromatogram; however, the remaining six steviol glycosides were not detected. Therefore, this method is not suitable for the simultaneous analysis of the nine steviol glycosides (Figure 2d). In summary, these four Ministry of Food and Drug Safety (MFDS) methods were unsuitable for the pretreatment of fermented milk, because they did not yield a desirable peak resolution. Therefore, it is necessary to review and improve the sample pretreatment method.

In the fifth method, we evaluated the use of NaH_2_PO_4_, which is a component in the mobile phase for HPLC analyses, for removing proteins and fat from the fermented milk samples. In addition, 50% MeOH was added for better solubility, and the solvent extraction mixture comprised 10 mM NaH_2_PO_4_:MeOH (1:1, *v*/*v*). An optimal sample pretreatment method was included to increase the recovery rate of steviol glycosides, which involved removing the fat and emulsification components and adjusting the pH of the HPLC mobile phase to 2.6. This method has the advantage of being simple (Figure 1), and it allowed all nine steviol glycosides to be identified (Figure 2e).

### 3.2. Separation of Steviol Glycosides

**Figure 3:**
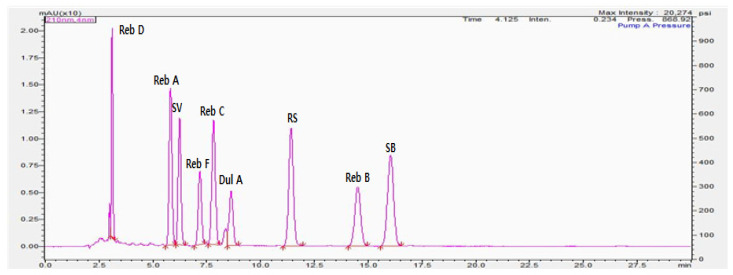
Chromatographic profile of steviol glycoside standards. Rebaudioside D (Reb D), rebaudioside A (Reb A), stevioside (SV), rebaudioside F (Reb F), rebaudioside C (Reb C), dulcoside A (Dul A), rubusoside (RS), rebaudioside B (Reb B), and steviobioside (SB).

Reb D, Reb A, SV, Reb F, Reb C, Dul A, RS, Reb B, and SB were eluted at retention times of 3.103, 5.918, 6.343, 7.327, 7.981, 8.811, 11.655, 14.891, and 16.432 min, respectively. The pretreatment method developed in this study allowed for the separation and detection of the nine steviol glycosides within 17 min. In particular, Reb A and SV, which are the major components of steviol glycosides, were detected within 6.4 min, which is in line with the results of other studies (<6.15 min; Figure 3) [21,34].

### 3.3. Calibration Curves, LOD, and Quantification

**Figure 4:**
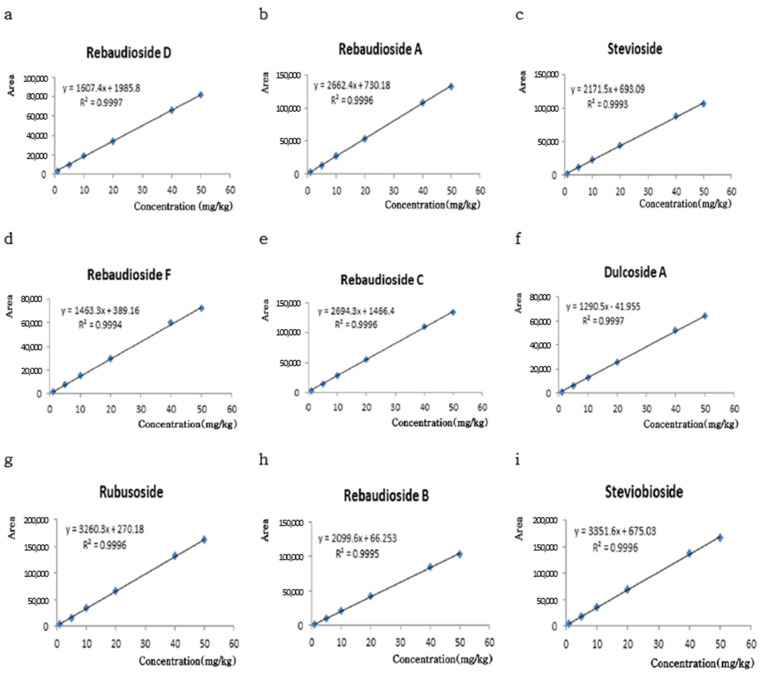
Calibration curve of the dependence of a peak area on the steviol glycoside concentration. (**a**) Rebaudioside D; (**b**) Rebaudioside A; (**c**) Stevioside; (**d**) Rebaudioside F; (**e**) Rebaudioside C; (**f**) Dulcoside A; (**g**) Rubusoside; (**h**) Rebaudioside B; and (**i**) Steviobioside.

Linearity was calculated by using the area-under-the-peak values for the six concentrations, which were measured in triplicate. A graph of the signal that was produced as a function of the analyte concentration was constructed. Linear regression was calculated by using the method of least squares (Figure 4). The parameters of simple linear regression were calculated for each of the nine standards at six concentrations: 1, 5, 10, 20, 40, and 50 mg/kg. The least squares for the linear regression analysis showed a high coefficient of determination (r^2^ = 0.9993–0.9997). These values were linear over the concentration range studied, suggesting that the model is suitable for quantifying the glycoside content. The LOD for steviol was 0.11–0.56 (S/N = 3), whereas the LOQ was 0.33–1.69 (S/N = 10). Therefore, the developed method could quantify the steviol glycoside contents at both high and low concentrations (Table 1).

### 3.4. Accuracy

Recovery tests were performed by using a mixture of commercial steviol glycosides (spiked at 6.25, 12.5, and 25 mg/kg), non-fermented milk (average 6% protein and 9% fat; 100 mg each), and fermented milk (average 8.3% protein and 7.8% fat; 100 mg each). Good recovery of the steviol glycosides was observed in the non-fermented milk (83.57–104.84%), with a relative standard deviation (RSD) of 0.22–2.35%. The recovery ranged from 84.71 to 103.98%, with an RSD of 0.16–2.83% in fermented milk (Table 2). The best recovery values ranged from 103 to 106% for low-fat milk and from 87 to 97% for yogurt, according to RP-HPLC/fluorescence [45]. The accuracy (recovery 92–120%), repeatability (3.1–5.4%), and laboratory precision (4.0–8.4%) were high for the recovery of steviosides from commercially available yoghurts (3.8% fat; 100 mg each). The values obtained in the present study were comparable to those obtained in an earlier study [49].

To the best of our knowledge, this is the first study to analyze the steviol glycoside content in dairy products with high fat and protein contents. This simple pretreatment method could increase the extraction efficiencies and decrease experimental errors in future studies.

### 3.5. Monitoring Test for Various Foods

We monitored eight different fermented milks and one non-fermented milk, all with high fat and protein contents. The non-fermented milk sample had 6% protein and 9% fat per 100 mg, while the fermented milk samples had 2–22% protein (average 8.3%) and 1–19% fat per 100 mg (average 7.8% fat).

In the non-fermented milk, the Reb D content was 427.89 mg/L, the Reb A content was 1.34 mg/L, and the SV content was 5.68 mg/L. In the fermented milk samples, the Reb D content was 63.79–363.81 mg/L (average: 167.28 mg/kg), the Reb A content was 27.64–122.3 mg/L (average: 63.79 mg/L), the SV content was 18.43–21.62 mg/L (average: 20.07 mg/L), and the Reb C content was 1.83–3.87 mg/L (average: 2.85 mg/L) (Table 3). Reb D, Reb A, and SV were detected in all samples, whereas Reb C was detected only in two fermented milk samples. This indicates that Reb D, Reb A, and SV are used in high amounts in commercial dairy preparations because of their high levels of sweetness. These levels meet the JECFA standard for beverages, desserts, and yogurt, which is less than 500 mg/kg. They also meet the Codex Alimentarius standard for dairy-based desserts (less than 330 mg/kg) and the ADI regulation of 0–4 mg steviol per kg body weight.

Steviol glycoside content is not a mandatory label on all food products; therefore, it was impossible to compare these values with the actual content. However, we confirmed that steviol use was within the permitted legal standards in the commercially distributed dairy products.

In addition, the detectable amounts of steviol glycosides in nondairy products, such as tea, soy sauce, and soju, are 0–13.7, 0–104.6, and 0–270.6 mg/kg, with average values of 0.9, 12.8, and 130.7 mg/kg, respectively [38]. The values of steviol vary between different food matrices. The results from this study emphasize the need for precise determination methods and correct labeling for providing accurate information to consumers. In addition, these values can be used as a guideline for monitoring dairy products in the future.

## 4. Conclusions

In this study, a simple protocol for extracting steviol glycosides from dairy products was described. HPLC combined with the newly developed pretreatment method allows for the direct measurement of steviol equivalents in dairy products. The use of MeOH and NaH_2_PO_4_ allowed for a more accurate determination of the total amount of steviol equivalents in the samples. Furthermore, the pretreatment method enabled the evaluation of the steviol glycoside content with high accuracy, linearity, and precision. As such, this pretreatment method can be applied to fermented milk with low steviol glycoside content. This simple protocol could contribute to the development of policy decisions associated with the nutritional labeling of dairy products consumed worldwide.

## Figures and Tables

**Table 1:** Validation and monitoring test for steviol glycosides.

Standard	LOD ^1^ (mg/kg)	LOQ ^2^ (mg/kg)	r^2^	Linear Regression
Rebaudioside D	0.19	0.57	0.9997	y = 1607.4x + 1984.8
Rebaudioside A	0.18	0.55	0.9996	y = 2662.4x + 730.18
Stevioside	0.19	0.57	0.9993	y = 2171.5x + 693.09
Rebaudioside F	0.12	0.37	0.9994	y = 1463.3x + 389.16
Rebaudioside C	0.11	0.33	0.9996	y = 2694.3x +1466.4
Dulcoside A	0.56	1.69	0.9997	y = 1290.5x + 41.995
Rubusoside	0.13	0.41	0.9996	y = 3260.3x + 270.18
Rebaudioside B	0.33	1.00	0.9995	y = 2099.6x + 66.253
Steviobioside	0.19	0.57	0.9996	y = 3351.6x + 675.03
Range	0–50 mg/mL			

^1^ LOD, limit of detection. S/N = 3. ^2^ LOQ, limit of quantitation. S/N = 10.

**Table 2:** Recovery test on non-fermented and fermented milk.

**Spiked Level ^1^**	**Non-Fermented Milk**									
		Reb D	Reb A	SV	Reb F	Reb C	Dul A	RS	Reb B	SB
6.25 mg/kg	Recovery (%)	99.37	96.61	88.34	101.68	83.96	85.57	84.59	104.31	83.57
	RSD ^2^ (%)	1.86	0.86	0.97	1.45	1.45	2.35	0.46	0.83	0.78
	SD ^3^	1.85	0.83	0.85	1.48	1.22	2.01	0.39	0.86	0.65
12.5 mg/kg	Recovery (%)	99.58	104.84	90.63	95.70	87.75	94.05	90.56	88.28	87.14
	RSD (%)	1.23	0.22	0.79	1.24	1.27	1.10	0.22	0.54	0.44
	SD	1.23	0.23	0.71	1.19	1.12	1.03	0.20	0.47	0.38
25 mg/kg	Recovery (%)	100.84	98.79	101.20	101.26	91.54	88.74	90.55	91.84	89.60
	RSD (%)	1.45	1.58	0.98	0.77	1.17	0.48	1.41	1.37	0.59
	SD	1.47	1.56	0.99	0.78	1.07	0.43	1.28	1.26	0.53
**Spiked Level**	**Fermented Milk**									
		Reb D	Reb A	SV	Reb F	Reb C	Dul A	RS	Reb B	SB
6.25 mg/kg	Recovery (%)	99.47	96.11	88.53	100.09	85.57	86.31	87.03	103.87	84.71
	RSD (%)	0.42	0.93	0.20	1.43	1.66	0.78	2.83	0.37	1.19
	SD	0.41	0.89	0.18	1.43	1.42	0.67	2.46	0.39	1.01
12.5 mg/kg	Recovery (%)	100.02	103.98	91.34	95.50	88.63	95.04	90.41	88.59	88.17
	RSD (%)	1.88	0.77	1.32	0.29	0.86	1.67	0.52	0.41	1.02
	SD	1.89	0.80	1.21	0.28	0.77	1.59	0.47	0.36	0.90
25 mg/kg	Recovery (%)	101.15	98.70	99.92	100.07	91.94	88.27	90.93	91.67	89.14
	RSD (%)	0.69	0.67	1.12	1.51	0.50	1.07	0.42	0.16	0.45
	SD	0.70	0.66	1.12	1.52	0.46	0.94	0.38	0.15	0.40

^1^ Spiking levels of samples were 6.25, 12.5, and 25 mg/kg. ^2^ RSD, relative standard deviation. ^3^ Values are the mean ± SD of three replicates. Reb D, rebaudioside D; Reb A, rebaudioside A; SV, Stevioside; Reb F, rebaudioside F; Reb C, rebaudioside C; Dul A, dulcoside A; RS, rebaudioside; Reb B, rebaudioside B; SB, steviobioside.

**Table 3:** Estimation of the steviol glycoside contents in nine commercially available dairy products.

Sample Name		Reb D ^1^		Reb A ^2^		SV ^3^		Reb C ^4^	
		Tested Value (mg/L)	RSD ^5^	Tested Value (mg/L)	RSD	Tested Value (mg/L)	RSD	Tested Value (mg/L)	RSD
Non-fermented milk	T-1	427.89 ± 0.58	0.14	1.34 ± 0.05	4.01	5.68 ± 0.09	1.53		
Fermented milk	T-2	99.38 ± 1.20	1.19	32.93 ± 0.05	0.14	20.40 ± 0.34	1.65	3.87 ± 0.09	2.19
	T-3	204.94 ± 4.29	2.09	58.92 ± 0.11	0.18	21.62 ± 0.04	0.16		
	T-4	91.12 ± 0.32	0.35	120.65 ± 0.88	0.73	18.51 ± 0.22	1.19	1.83 ± 0.05	2.77
	T-5	217.23 ± 1.64	0.76	122.30 ± 0.98	0.80	21.03 ± 0.72	3.44		
	T-6	363.81 ± 3.82	1.05	43.00 ± 0.18	0.43	18.43 ± 0.23	1.25		
	T-7	124.16 ± 1.31	1.05	55.10 ± 0.33	0.59	20.79 ± 0.47	2.26		
	T-8	173.81 ± 2.74	1.57	27.64 ± 0.29	1.05	20.68 ± 0.32	1.56		
	T-9	63.79 ± 1.09	1.72	49.85 ± 0.13	0.27	19.47 ± 0.34	1.73		

^1^ Reb D, rebaudioside D; ^2^ Reb A, rebaudioside A; ^3^ SV, stevioside; ^4^ Reb C, rebaudioside C; ^5^ RSD (%), relative standard deviation.

## Data Availability

Not applicable.
